# The association between travel time to health facilities and childhood vaccine coverage in rural Ethiopia. A community based cross sectional study

**DOI:** 10.1186/1471-2458-12-476

**Published:** 2012-06-22

**Authors:** Yemisrach B Okwaraji, Kim Mulholland, JoannaRMArmstrong Schellenberg, Gashaw Andarge, Mengesha Admassu, Karen M Edmond

**Affiliations:** 1 Faculty of Epidemiology and Population Health, London, School of Hygiene & Tropical Medicine, London, UK; 2 Faculty of Infectious and Tropical Diseases, London School of Hygiene & Tropical Medicine, London, UK; 3 Institute of Public Health, College of Medicine and Health Sciences University of Gondar, Gondar, Ethiopia; 4 President’s office, University of Gondar, Gondar, Ethiopia; 5 School of Paediatrics and Child Health, University of Western Australia, Perth, Western Australia

## Abstract

**Background:**

Few studies have examined associations between access to health care and childhood vaccine coverage in remote communities that lack motorised transport. This study assessed whether travel time to health facilities was associated with childhood vaccine coverage in a remote area of Ethiopia.

**Methods:**

This was a cross-sectional study using data from 775 children aged 12–59 months who participated in a household survey between January –July 2010 in Dabat district, north-western Ethiopia. 208 households were randomly selected from each *kebele*. All children in a household were eligible for inclusion if they were aged between 12–59 months at the time of data collection. Travel time to vaccine providers was collected using a geographical information system (GIS). The primary outcome was the percentage of children in the study population who were vaccinated with the third infant Pentavalent vaccine ([Diphtheria, Tetanus,-Pertussis Hepatitis B, *Haemophilus influenza* type b] Penta3) in the five years before the survey. We also assessed effects on BCG, Penta1, Penta2 and Measles vaccines. Analysis was conducted using Poisson regression models with robust standard error estimation and the Wald test.

**Results:**

Missing vaccination data ranged from 4.6% (36/775) for BCG to 16.4% (127/775) for Penta3 vaccine. In children with complete vaccination records, BCG vaccine had the highest coverage (97.3% [719/739]), Penta3 coverage was (92.9% [602/648]) and Measles vaccine had the lowest coverage (81.7% [564/690]). Children living ≥60mins from a health post were significantly less likely (adjRR = 0.85 [0.79-0.92] p value < =0.001) to receive Penta3 vaccine compared to children living <30mins from a health post. This effect was not modified by household wealth (p value = 0.240). Travel time also had a highly significant association with BCG (adjRR = 0.95 [0.93-0.98] p value =0.002) and Measles (adjRR = 0.88 [0.79-0.97] p value =0.027) vaccine coverage.

**Conclusions:**

Travel time to vaccine providers in health posts appeared to be a barrier to the delivery of infant vaccines in this remote Ethiopian community. New vaccine delivery strategies are needed for the hardest to reach children in the African region.

## Background

Vaccines are one of the most effective interventions that can be used to prevent mortality in children under five years [1]. Improving access to childhood vaccines in low-income countries has been a major goal of public health services both at international and national levels [1]. However, achieving high and equitable coverage remains a challenge in low income countries like Ethiopia [2].

Ethiopia has implemented the World Health Organization (WHO) Expanded Programme on Immunisation (EPI) schedule since 1980 [2]. The EPI now targets eight diseases: Tuberculosis, Poliomyelitis, Diphtheria, Tetanus, Pertussis, Hepatitis B, *Haemophilus influenzae* type b and Measles and prescribes eight vaccines to be administered to children in their first year of life [3]. UNICEF and WHO have prioritised vaccination of children from the “hardest to reach” populations including those with limited geographical access to health facilities [4] and programs such as ‘Reaching Every District’ (RED) and ‘National Immunisation Days (NIDs)’ have been introduced in many countries including Ethiopia [5]. These programs appear to have improved access to vaccines across Ethiopia. However, over one third of families in rural Ethiopia still have to walk more than 60 minutes to reach health posts for vaccines [6].

In response to these concerns Ethiopia’s health sector reform plan has included a Health Extension Program to improve access and equity to health interventions including immunisation services for the rural poor [7]. This has included the staffing of health posts in remote areas to provide basic services such as health education and childhood immunisation. The Health Extension Program has improved quality and reduced travel time to vaccination services and may have reduced many barriers to the implementation of vaccine programs in remote areas. However, the impact of these programs in the poorest and most remote regions is unknown. The primary objective of this study was to assess whether travel time to health posts was associated with childhood vaccine coverage in a remote area of rural Ethiopia. Secondary objectives were to assess if vaccination coverage varied by household wealth status and if the effect of travel time on vaccine coverage was modified by household wealth.

## Methods

### Study area

The study was implemented in the Dabat Health and Demographic Surveillance Site (HDSS) in Dabat district, north-western Ethiopia. The HDSS consists of three urban and seven rural *kebeles* - the smallest administrative unit in Ethiopia. The population in the HDSS is currently 46,165 and is dominated by the Amhara ethnic group [8]. A typical house has walls constructed from mud and wood. Livestock are commonly kept in the house and the economy is mainly based on subsistence farming and trading. There are few roads in this part of Ethiopia. Off-road motorised transport is not viable in most areas because of the difficult terrain. The main form of travel is walking and the mountainous region and poor road network means that families spend many hours walking to farms, health facilities and administrative centres. The under five mortality rate in Dabat district has recently been reported as 130 per 1,000 live births [9].

The vaccine schedule in Dabat district comprises: Bacille Calmette Guérin (BCG); Pentavalent vaccine (Diphtheria, Tetanus, Pertussis, Hepatitis B and *Haemophilus influenzae* type b vaccines combined in one syringe) at 6 weeks (Penta 1), 10 weeks (Penta2) and 14 weeks (Penta3); oral polio vaccine (OPV) at 6 weeks (Polio1), 10 weeks (Polio2) and 14 weeks (Polio3) and measles vaccine at 9 months.

There are a total of 8 rural health posts in Dabat district (one *kebele* has two health posts and all other rural *kebeles* have one health post). In Dabat district, vaccines are mostly administered in the health posts. Health posts are the lowest level of health care and staffed by two female health extension workers recruited from the community and trained for one year to provide a range of essential health interventions including childhood immunisation for the rural population [10]. Vaccines are provided approximately once a month to all children attending the health post [3]. Due to limited time and staffing the health extension workers do not perform home visits to provide vaccines. There are no refrigerators to keep the vaccines in the health posts and they are brought in a vaccine carrier from Dabat health centre on the scheduled date for immunisation and left over vaccines are taken back to the health centre the same day. The other source of vaccine delivery in the study area is the national immunisation days. These are currently used for Polio and Measles vaccines and are implemented by national immunisation staff in community locations (e.g. village market places, schools, churches or mosques) as well as the health posts.

### Study design

This was a cross-sectional study conducted in the seven rural *kebeles* of the HDSS between January and July 2010. 208 households were randomly selected from a list of the rural *kebele* households supplied by the HDSS using a computer generated sequence. Children were eligible for inclusion if they were aged between 12–59 months at the time of data collection.

### Data collection

Trained data collectors visited households and identified eligible children who were present at the time of the survey. Information recorded during the interview included: demographic characteristics of mothers, household asset information needed for the construction of wealth terciles, the place of the child’s birth and whether delivery was assisted by skilled birth attendants. After obtaining informed written consent from the child’s mother, the data collectors asked the mothers questions and entered the responses directly into the hand-held computers (PDAs). Vaccination data for five vaccines (BCG, Penta1, Penta2, Penta3 and Measles) were recorded from the vaccine card obtained from the mother at the beginning of the interview. If there was no card, data were obtained from the mother, based on her memory about the vaccination. Immunisation data were double checked from the health post vaccination register book and information was added or corrected where needed. Polio vaccination data were not recorded as both maternal recall and health post registration for these vaccines was very poor.

### Geographical access data

Methods for calculation of the measures of geographical access are detailed elsewhere [9]. In brief, travel time was calculated using the “Cost analysis” module in the IDRISI Taiga Geographic Information System (GIS) software package. The module requires two input layers of data. The first layer contains the target location (the health posts), and the second layer contains the costs (in terms of the time spent walking) associated with moving through different geographical features in the study area to reach the target feature. Different features (e.g. walking up hills and mountains and traversing through water) are assigned different speeds. The output from the module is an image where each cell (pixel) in the image contains values of travel time required to traverse from that cell to the health post. In this study, a speed of 5 km/hr was assigned for all walking routes, slopes greater than 30 degrees were assigned a speed of 0.1 km/hr and traversing through water bodies was also assigned a speed of 0.1 km/hr. Travel time for each household was extracted and exported into Stata to merge with the main dataset. Validation of the model was described in detail in our previous paper [9]. In brief, reported travel time from 40 village centre were obtained and compared with estimated travel time. Mean reported and estimated travel times were very close (mean, 73 vs. 67 minutes; standard deviation, 46 vs. 40 minutes, respectively).

### Statistical methods

All analyses were performed in Stata SE 12.0 (StataCorp LP, College Station, TX 77845, USA). Travel time was examined as a categorical variable divided into terciles (<30mins, 30- < 60mins and ≥60 mins) and as a continuous variable (based on the number of minutes of travel time from the household to the health post).

The primary outcome variable was the percentage of children aged 12–59 months who received Penta3 vaccine at any time before the survey was conducted. This vaccine was chosen as it is most commonly used by international groups such as WHO and UNICEF [11].

To estimate the wealth status of each household, we constructed a relative asset index based on data collected on housing material and household assets. The index was constructed using principal component analysis (PCA). Children in the sample were ranked in order of the asset index values for their households, and then they were divided into one of three equal sized terciles, ranking from the least poor to the poorest.

In the univariable analyses, we first assessed associations between Penta3 coverage, travel time, household wealth and potential confounding variables such as: demographic characteristics of mothers (age group, education level, and parity). In the multivariable analysis we investigated the relationship between the probability of receiving Penta3 vaccine and travel time to health posts using Poisson regression models with robust standard error estimation. We first accounted for the intra cluster correlation of many children from the same mother, many mothers from the same village and many villages from the same kebele. However, there was only evidence of within kebele clustering of vaccination coverage and no evidence of clustering at the village or mother level. Thus, the final model only adjusted for clustering at the kebele level. The model also included confounding variables which had p value of <0.1 for their association with travel time to health posts and vaccine coverage. We also examined statistical interactions between travel time and household wealth status and hypothesised a priori that the effect of travel time on vaccine coverage might be different in rich and poor women. The Wald test was used to compare the fit of models containing different variables, to test for trend in ordered categorical variables and to test for a statistical interaction between travel time and household wealth. These analyses were repeated for the four other vaccines (BCG, Penta1, Penta2 and Measles).

### Sample size

The most recent Ethiopian Demographic and Health Survey (DHS) reported 32% of children aged 12–59 months are immunised with Penta3 vaccine by age 12 months [12]. The Dabat HDSS reported that there were approximately 600 children aged 12–59 months in the study area. We calculated that these children would provide 80% power at a 5% significance level to detect at least a 10% difference in Penta3 vaccination coverage between children who lived < 60 minutes from a health post compared to those who lived ≥ 60 minutes.

### Ethical considerations

This study was approved by the ethical review committees of the University of Gondar and the London School of Hygiene and Tropical Medicine. Verbal informed consent to conduct interviews was received from all heads of participating households. Written informed consent to participate in the study was obtained from mothers of all children who were included in the study.

## Results

### Study population

**Figure 1:**
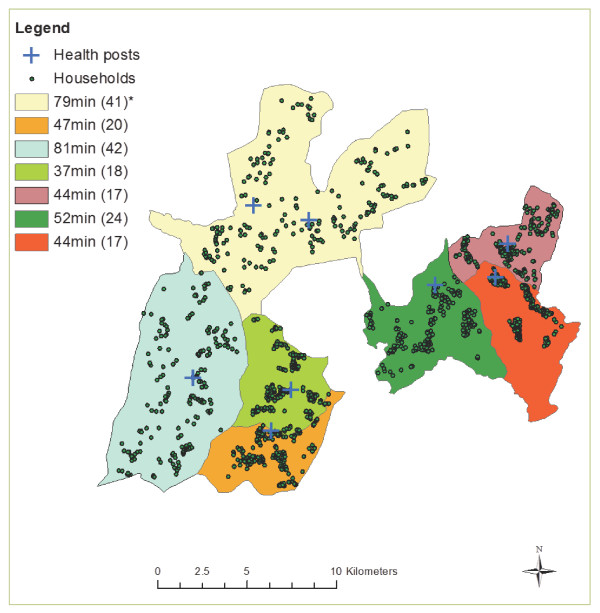
**Mean travel time to health posts by location of kebeles in Dabat, rural Ethiopia.** * Mean travel time in the kebele (standard deviation).

We visited 1,453 households in 7 rural *kebeles*. All households were visited and all women in these households were able to be interviewed. 775 children were aged between 12–59 months. Of these, 380 (49%) were female; 494 (65%) lived <60 mins walking travel time from the health post; the overall mean travel time was 55 mins (standard deviation [sd] 33 mins) and the median was 51 mins (interquartile range [IQR] 0-168mins). The mean travel time by location of kebele ranged from 37–81 mins (Figure 1). 355 (53%) of the mothers were aged over 30 years. The mean number of children per woman was 4.0 (sd 2.1) and the mean age of the mothers interviewed was 29 years (sd 7.1). 280 (37%) children lived in the poorest and 203 (27%) in the least poor households. Only 17 (2.1%) were born at a health facility and 16 (2.0%) of births were attended by a skilled birth attendant.

**Figure 2:**
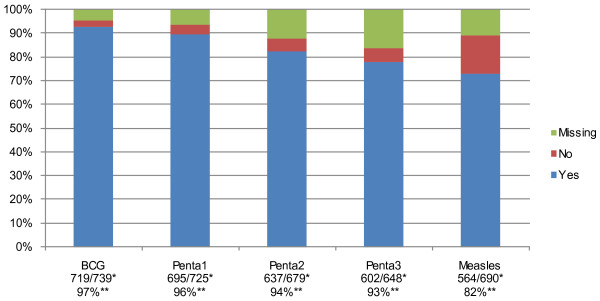
**Vaccine coverage in children aged 12–59 months in Dabat, rural Ethiopia.** *Number of children who received vaccine / number of children with vaccination data (excluding those with missing vaccination data). **Vaccine coverage (%).

Figure 2 summarises vaccination coverage for the five vaccines in children aged 12–59 months. Missing vaccination data ranged from 4.6% (36/775) for BCG to 16.4% (127/775) for Penta3 vaccine. In children with complete vaccination records, BCG vaccine had the highest coverage (97.3% [719/739]), Penta3 coverage was (92.9% [602/648]) and Measles vaccine had the lowest coverage (81.7% [564/690]).

### Socio demographic characteristics

Table 1 shows the univariable analysis of association, in children with complete vaccination records, between vaccination coverage and child’s sex, location, mother’s education, mother’s age and parity for each of the vaccines. There was little variation in vaccination coverage by child’s sex, mother’s age or education level. However, there was marked variation by *kebele*.

**Table 1:** Vaccine coverage in children with complete vaccination records aged 12–59 months, by sociodemographic characteristics in Dabat, rural Ethiopia

**Variables**	**Categories**	BCG n = 719*	**Penta 1 n = 695**	**Penta 2 n = 637**	**Penta 3 n = 602**	**Measles n = 564**
**Sex**	Boys	342(96%)**	337(96%)	316(95%)	299(94%)	272(81%)
	Girls	354(98%)	335(95%)	302(92%)	285(92%)	277(83%)
	Missing data	23	23	19	18	15
	*p-value*	*0.162*	*0.277*	*0.095*	*0.237*	*0.449*
**Location**	Kebele1	93(97%)	52(68%)	40(62%)	31(86%)	68(82%)
	Kebele2	123(99%)	123(99%)	117(99%)	114(99%)	96(85%)
	Kebele3	121(100%)	121(100%)	120(99%)	120(99%)	94(81%)
	Kebele4	87(99%)	89(95%)	69(97%)	67(93%)	76(83%)
	Kebele5	98(94%)	104(100%)	100(97%)	89(86%)	75(80%)
	Kebele6	112(92%)	121(00%)	107(91%)	97(84%)	84(71%)
	Kebele7	85(100%)	85(100%)	84(99%)	84(98%)	71(96%)
	Missing data	-	-	-	-	-
	*p-value*	*0.001*	*<=0.0001*	*<=0.0001*	*<=0.0001*	*0.003*
**Mother’s education**	Education	110(100%)	100(95%)	94(94%)	92(95%)	91(85%)
	No education	587(97%)	573(96%)	525(94%)	493(93%)	459(82%)
	Missing data	22	20	18	17	14
	p-value	*0.053*	*0.786*	*0.975*	*0.259*	*0.422*
**Mother’s age**	16- < 25y	147(98%)	138(95%)	127(91%)	117(87%)	109(79%)
	25- < 30y	184(96%)	182(94%)	168(93%)	158(95%)	149(83%)
	30- < 35y	163(99%)	156(96%)	142(96%)	139(96%)	130(85%)
	35- < 50y	203(96%)	197(97%)	182(96%)	171(93%)	162(81%)
	Missing data	22	22	18	17	14
	*p-value*	*0.188*	*0.446*	*0.162*	*0.019*	*0.503*
**Parity**	1	56(98%)	52(93%)	51(93%)	47(90%)	42(82%)
	2-3	216(99%)	204(95%)	187(92%)	177(93%)	166(82%)
	4-5	215(96%)	207(94%)	189(93%)	177(92%)	167(83%)
	6-12	210(96%)	210(98%)	192(96%)	184(94%)	175(82%)
	Missing data	22	22	18	17	14
	*p-value*	*0.392*	*0.074*	*0.347*	*0.768*	*0.989*

* Total number of children who were vaccinated.

** Number of children vaccinated (number of children vaccinated / number of children with vaccination data).

### Travel time

**Figure 3:**
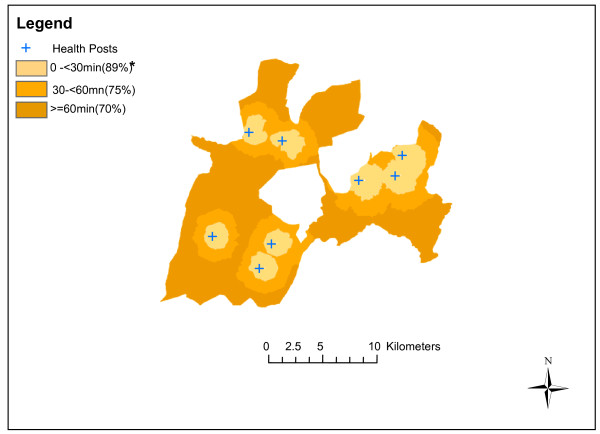
**Proportion of children aged 12–59 months who received Penta3 vaccination by travel time to health posts in Dabat, rural Ethiopia.** * Mean travel time (vaccine coverage, %).

Table 2 displays the Poisson regression analysis for both the crude and adjusted assessment of the effect of travel time to health posts on the risk of being vaccinated against BCG, Penta1, Penta2, Penta3 and Measles. Travel time appeared to have a highly significant association with Penta3 vaccine coverage. Children living ≥60mins from a health post were significantly less likely (adjRR 0.85 [0.79-0.92] p-value < =0.0001) to receive Penta3 vaccine compared to children living <30mins from a health post (Table 2 and Figure 3). Travel time also had a highly significant association with BCG (adjRR = 0.95 [0.93-0.98] p value =0.002) and Measles, (adjRR = 0.88 [0.79-0.97] p value =0.027) vaccine coverage.

**Table 2:** Poisson regression analysis of the association between travel time to health posts and vaccine coverage in children with complete vaccination records aged 12–59 months in Dabat, rural Ethiopia

**Variables**	**Travel time**	**Vaccinated (%)**	**Crude RR (95%CI)**	Adjusted RR (95%CI)**	P-value***
BCG(n = 739)*	0- < 30 min	241(97%)	1	1	-
	30- < 60mins	240(94%)	0.98(0.97-1.00)	0.98(0.97-1.00)	0.071
	> = 60mins	238(86%)	0.95(0.93-0.98)	0.95(0.93-0.98)	0.002
**Penta 1(n = 725)**	0- < 30 min	238(96%)	1	1	-
	30- < 60mins	217(85%)	0.93 (0.84-1.04)	0.93 (0.84-1.04)	0.234
	> = 60mins	240(88%)	0.95(0.86-1.04)	0.95(0.86-1.04)	0.275
**Penta 2(n = 679)**	0- < 30 min	223(90%)	1	1	-
	30- < 60mins	199(78%)	0.92(0.82-1.04)	0.92(0.82-1.04)	0.197
	> = 60mins	215(78%)	0.94(0.89-0.99)	0.94(0.89-0.99)	0.030
**Penta 3(n = 648)**	0- < 30 min	220(89%)	1	1	-
	30- < 60mins	191(75%)	0.96(0.94-1.00)	0.92(0.89-0.96)	0.034
	> = 60mins	191(70%)	0.85(0.79-0.92)	0.85(0.79-0.92)	<0.0001
**Measles(n = 690)**	0- < 30 min	198(80%)	1	1	-
	30- < 60mins	188(74%)	0.95(0.89-1.02)	0.95(0.89-1.02)	0.214
	> = 60mins	178(65%)	0.88(0.79-0.97)	0.88(0.79-0.97)	0.027

*Total number of children with complete vaccination records.

**Adjusted for household wealth, mother’s education, parity and mother’s age.

***P values from Wald test.

Adjusting for household wealth, mother’s age, education, and parity had little effect on all effect measures in the multivariable analyses.

### Household wealth

Table 2 displays the Poisson regression analysis for both the crude and adjusted analysis of the effect of household wealth on the risk of being vaccinated against BCG, Penta1, Penta2, Penta3 and Measles. There appeared to be no association between household wealth and coverage of any vaccine. There was also no statistical evidence of modification of the effect of travel time on Penta3 vaccine coverage by household wealth (p value = 0.240) or on any other vaccine (Table 3).

**Table 3:** Poisson regression analysis of the association between household wealth and vaccine coverage in children with complete vaccination records aged 12–59 months, in Dabat, rural Ethiopia

**Variables**	**Household wealth**	**Immunised (%)**	**Crude RR (95%CI)**	Adjusted RR (95%CI)**	P-value***
BCG(n = 739)*	Least poor	182(90%)	1	1	-
	Middle	249(92%)	1.01(0.96, 1.07)	1.02(0.95,1.07)	0.643
	Poorest	266(95%)	1.03(0.98,1.08)	1.03(0.98,1.08)	0.152
**Penta 1(n = 725)**	Least poor	172(85%)	1	1	-
	Middle	245(91%)	1.00(0.98,1.03)	1.01(0.98,1.04)	0.463
	Poorest	256(91%)	0.99(0.94,1.05)	1.00(0.94,1.07)	0.756
**Penta 2 (n = 679)**	Least poor	162(80%)	1	1	-
	Middle	224(83%)	0.96(0.91,1.03)	0.96(.091,1.03)	0.409
	Poorest	233(83%)	0.97(0.93,1.02)	0.97(0.93,1.02)	0.728
**Penta 3 (n = 648)**	Least poor	151(74%)	1	1	-
	Middle	212(78%)	1.03(0.97,1.10	1.04(0.98,1.10)	0.221
	Poorest	222(79%)	1.01(0.96,1.06)	1.02(0.97,1.08)	0.414
**Measles (n = 690)**	Least poor	146(72%)	1	1	-
	Middle	202(75%)	0.98(0.89,1.08)	0.98(0.89,1.08)	0.720
	Poorest	202(72%)	0.96(0.89,1.08)	0.96(0.89,1.04)	0.402

*Total number of children with complete vaccination records.

**Adjusted for travel time to health posts, mother’s education, parity and mother’s age.

***P values from Wald test.

## Discussion and Conclusions

Travel time to vaccine providers in health posts appeared to be a barrier to the delivery of infant vaccines in this remote community. However, there appeared to be no differential or modification of this effect by household wealth. We recently reported that geographic access to the major health centre in the study area was an important determinant of both early and late child mortality [9,13-15]. Our analyses now indicate that travel time also effects the implementation of preventative interventions such as infant vaccination.

Our findings were clearly defined for all vaccines, though the effect was strongest for pentavalent vaccine. This may be due to the fact that pentavalent vaccines are only delivered at the static health posts while Measles vaccines are given through supplemental immunisation activities [16-18] within the villages and BCG vaccines are given at the time of delivery.

This appears to be the first study that has examined associations between geographical access to health facilities and childhood vaccination coverage throughout infancy in such a remote area of Africa. This also appears to be the first study that reports that Measles vaccination may be less effected by geographic disparities than other vaccines. Studies from Tanzania [19], Malawi [20], Nigeria [21], Kenya [22] and Papua New Guinea [23], all indicated that travel time to health facilities was a barrier to receipt of all infant vaccines including Measles [23]. Studies from more densely populated areas such as Kenya found no association between the coverage of any infant vaccines and travel time to health facilities [24-26]. This was thought to be due to the high density of health centres and health posts rather than the effect of supplementary immunisation activities or other Ministry of Health strategies.

Interestingly there was a marked association between vaccination coverage and *kebele.* This may be due to differences in quality of services or other barriers such as social constraints or staffing levels in the different *kebeles*. However, we did not adjust the results for travel time or other potential confounding factors and further analyses are needed to explore this issue. There was also little variation in vaccination coverage by household wealth, maternal age or education. This is likely to be due to the marked homogeneity in our study population and we reported similar effects in our earlier mortality study [9].

There were several limitations to our study. Firstly, we were not able to assess coverage of important vaccines such as Polio due to problems with reporting. Secondly, we were only able to assess effects on vaccination coverage and could not examine effects on health outcomes such as vaccine preventable diseases or hospitalisations due to limited resources. Thirdly, as this was a cross sectional study, travel time was assigned at the point at which the mothers were interviewed and there was no ongoing tracking of migration. Thus there may have been some misclassification of travel time status. Fourthly, the timeliness of the vaccination was not evaluated in this study as mothers recall was considered to be too poor. Finally, we may have overestimated the percentage of vaccinated children as we only included surviving children in our study and we might have missed unvaccinated children who had died [27,28]. In addition, our coverage data were calculated using children aged 12–59 months and are likely to be higher than other studies and the DHS which use younger children aged 12–23 months [29]. Five to seventeen percents of vaccine data was also unknown or missing in our study, however, any differential misclassification of vaccination status would have tended to underestimate rather than overestimate effect sizes. Also, important strengths of our study were our careful collection of health facility data on vaccination and our reporting of potential confounders such as maternal education and household wealth. We also used a three-level-level random effects model to account adequately for clustering by mother, village and kebele. We also used a validated access measure [30] (travel time) from each household to the health posts which accounted for the influence of topography and other natural barriers.

Our study has important implications for policy and program development. Vaccination coverage can be improved in remote areas by improving access and reducing travel time to health facilities. Supplementary immunisation activities may also be contributing to improvements in measles immunisation coverage in later infancy and reduction in inequalities. Where appropriate, outreach programs and supplemental immunisation activities should be considered for raising coverage in remote areas. In addition, new vaccine delivery strategies are needed for the hardest to reach children in Ethiopia and other parts of sub-Saharan Africa.

## Competing interests

The authors declare that they have no competing interests.

## Authors’ contributions

Conceived and designed the experiments: YO KE KM. Performed the experiments: YO. Analyzed the data: YO. Contributed materials/analysis tools: YO KE JO KM GA MA. Wrote the paper: YO KE. Edited the manuscript: JO KM GA MA. All authors read and approved the final manuscript.

## Pre-publication history

The pre-publication history for this paper can be accessed here:

http://www.biomedcentral.com/1471-2458/12/476/prepub
